# Effectiveness of Problem-Based Learning versus Traditional Teaching Methods in Improving Acquisition of Radiographic Interpretation Skills among Dental Students—A Systematic Review and Meta-Analysis

**DOI:** 10.1155/2021/9630285

**Published:** 2021-09-24

**Authors:** Alexander Maniangat Luke, Simy Mathew, Sam Thomas Kuriadom, Jeny Mary George, Mohmed Isaqali Karobari, Anand Marya, Ajinkya Mansing Pawar

**Affiliations:** ^1^Department of Clinical Sciences, College of Dentistry, Ajman University, Al-Jurf, Ajman, UAE; ^2^Center of Medical and Bio-Allied Health Sciences Research, Ajman University, Ajman, UAE; ^3^Department of Basic Medical and Dental Sciences, Ajman University, College of Dentistry, Ajman 346, UAE; ^4^Conservative Dentistry Unit, School of Dental Sciences, Universiti Sains Malaysia, Health Campus, 16150 Kubang Kerian, Kota Bharu, Kelantan, Malaysia; ^5^Department of Conservative Dentistry & Endodontics, Saveetha Dental College & Hospitals, Saveetha Institute of Medical and Technical Sciences University, Chennai, 600077 Tamil Nadu, India; ^6^Department of Orthodontics, Faculty of Dentistry, University of Puthisastra, Phnom Penh 12211, Cambodia; ^7^Department of Orthodontics, Saveetha Dental College & Hospitals, Saveetha Institute of Medical and Technical Sciences University, Chennai, 600077 Tamil Nadu, India; ^8^Department of Conservative Dentistry and Endodontics, Nair Hospital Dental College, Mumbai, 400034 Maharashtra, India

## Abstract

Problem-based learning is an experiential and student-centred learning method to practice important skills like querying, critical thinking, and collaboration through pair and group work. The study is aimed at comparing the effectiveness of problem-based learning (PBL) and traditional teaching (TT) methods in improving acquisition of radiographic interpretation skills among dental students. Clinical trials (randomized and nonrandomized) were conducted with the help of dental students studying oral radiology using PBL and TT methods and assessing radiographic interpretation skills, knowledge scores, and satisfaction level as outcomes. Articles published from PubMed/MEDLINE, DOAJ, Cochrane Central Register of Controlled Trials, and Web of Science were searched. The quality of the studies was evaluated using the Cochrane Collaboration Tool, the MINORS Checklist, and the Risk of Bias in Nonrandomized Studies of Interventions (ROBIN-I) tool. Meta-analysis was done using Review Manager 5.3. There were twenty-four articles for qualitative synthesis and 13 for meta-analysis. The cumulative mean difference was found to be 0.54 (0.18, 0.90), 4.15 (-0.35, 8.65), and -0.14 (-0.36, 0.08) for radiographic interpretation skills, knowledge scores, and satisfaction level, respectively, showing significant difference favouring PBL as compared to TT except for satisfaction level which favoured the TT group. To understand the long-term effectiveness of PBL over TT methods in oral radiology among dental students, well-designed long-term randomized controlled trials are needed.

## 1. Introduction

Knowledge transfer is a major concern in improving educational practices. “Knowledge” is categorized into three areas by the cognitive psychologists as “declarative knowledge,” “procedural knowledge,” and an ill-defined grey zone between declarative and procedural knowledge that includes the reasoning skills often described as critical thinking and problem solving [1]. Traditional teaching methods often follow linear or modular learning, which is a highly directed, controlled, and program-centred approach as directed by the tutor, wherein the learners complete the given activities without developing the critical reasoning skill [2].

To develop problem-solving ability, one must progress from convergent thinking to critical thinking by adopting a learner-centric approach, emphasizing on self-directed learning and designing interlinkable yet independent resources that the learner can explain in his or her own words [3]. Problem-based learning (PBL) is an educational approach in which a problem serves as an incentive toward finding solutions leading to active, self-motivated, and dynamic education [4]. PBL was introduced into health sciences education at McMaster University in 1969 and was first introduced to dental education at the Faculty of Odontology in Malmö, Sweden, in 1990 [5].

The core model of PBL (see the overview of Barrows in 1996) is composed of the following six characteristics: learning is student centred, learning occurs in small student groups, tutors are facilitators or guides, problems form the organizing focus and stimulus for learning, problems are a vehicle for the development of problem-solving skills, and new information is attained through self-directed learning [6].

Oral radiology is an indispensable part of undergraduate and postgraduate dental training. Radiographs form an essential diagnostic tool for patient assessment and treatment planning and form the mainstay of all clinical specialties of dentistry [7]. Learning the basic skills of interpretation of intra- and extraoral radiographs requires having (1) mastery in perception, which is the ability to recognize abnormal patterns on a radiograph, and (2) cognition, i.e., the ability to interpret these abnormal patterns to arrive at a diagnosis which are the two distinguishable and inseparable components of visual diagnosis [8].

Traditionally, during the clinical posting of Oral Radiology, the dental students are taught about skills of radiographic interpretation in a batch of 10–12 students by the tutor. This type of learning is passive and teacher-centred, and therefore, the students may develop a minimal capacity for adopting a deep approach to learning, searching for deeper meaning and personal relevance in the topic and are therefore unable to apply learned concepts in new situations competently [9].

The emergence of newer nonlinear teaching and learning methods such as action-based learning, competency-based education, contextual- and inquiry-based learning, lifelong learning, problem-based learning, and self-directed learning showed a tremendous increase of educational literature on conduction and implementation of these new learning appraches [10]. In the same way, oral radiography teaching has been going through a renovation from the traditional didactic system to an approach of problem-based learning whereby students take a more active role in their learning. The flipped classroom (FC) model is an integrated method for learning in which students review content ahead of the classroom session and teachers use class time for active learning [11]. Implementation of syndicate-based learning [9] and the one-minute preceptor (OMP) model provide experiential learning to the students for future practice [12]. Radiology simulator-supported training [13] and digital environments allow visual communication between the educator and the learner, thus promoting appealing and engaging participation and better understanding [14]. The above studies on problem-based learning, as compared to traditional teaching methods, have reported acceptable positive gains in cognitive outcomes.

There are few systematic reviews in dentistry that have compared the effectiveness of PBL with traditional teaching approaches across various specialties of dentistry, but none have been specifically done to assess the effectiveness of these newer approaches in the field of oral medicine and radiology. The aim of this systematic review is to summarize the evidence and compare the effectiveness of PBL with that of traditional teaching approaches in improving acquisition of radiographic interpretation skills among dental students. The null hypothesis is as follows: “There is no difference in PBL and traditional teaching approaches in improving acquisition of radiographic interpretation skills among dental students.”

## 2. Methods

This systematic review and meta-analysis are written and reported according to the Preferred Reporting Items for Systematic Review and Meta-Analyses (PRISMA) statement and registered in PROSPERO under number CRD42020184441. The proposed focused research question in the Patient, Intervention, Comparison, and Outcome (PICO) format is as follows: “Is there a difference in the effectiveness of problem-based learning (PBL) versus traditional teaching methods (TT) in improving acquisition of radiographic interpretation skills among dental students?”

### 2.1. Search Strategy

A comprehensive electronic search was carried out by two of the authors independently (STK, JG) on databases, such as PubMed/MEDLINE, Cochrane Central Register of Controlled Trials, and Web of Science until June 2020 to retrieve articles in the English language. A specific electronic search of journals presented in Table 1 was conducted. The searches in the clinical trials database, cross-referencing, and grey literature were conducted using Google Scholar, Greylist, and OpenGrey. The complete process of the literature search is mentioned in Screening Process.

Medical Subject Heading (MeSH) terms, keywords, and other free terms combined with Boolean operators (OR, AND) were used for searching articles. The search strategy and population, interventions, comparisons, outcomes, and the study design (PICOS) tool are presented in Table 1.

### 2.2. Inclusion Criteria Outline according to the PICOS Strategy

- Population (P): studies reporting dental undergraduate and postgraduate students who are taught the subject of oral medicine and radiology
- Interventions (I): problem-based learning, case-based learning, syndicate learning, case-based blended learning, one-minute preceptor, small-group teaching, simulation teaching, virtual teaching, schema-based teaching, and active learning
- Comparison (C): dental students who receive other learning schemes that are traditional methods of teaching/non-problem-based learning-lectures, passive learning, and instructional learning
- Outcome (O): primary outcome—improved radiographic interpretation skills, knowledge, and student's perception; proficiency test of the dental students
- Secondary outcome: student perceptions towards PBL methods
- Study design (S): clinical trials, randomized controlled studies, nonrandomized control trials, quasiexperimental studies, before and after study design, and cohort studies comparing the effect of PBL and traditional training

Since RCTs are considered a gold standard of clinical trials, we intended to include those. Also, we included the other studies, as they are the most common study types which included human participants as their subjects. Ultimately, we aimed to summarize the evidence, and we tried to include all the types of study designs mentioned above for the same.

### 2.3. Exclusion Criteria

Review reports, case series, cross-sectional studies, and survey reports were excluded. The article which did not report the elements of PBL as described by Barrows in 1996 [6] was excluded. In addition, articles reporting about a single intervention were excluded. Articles reporting only abstracts were also excluded.

### 2.4. Screening Process

The search and screening according to the previously established protocol were conducted by two reviewing authors independently (STK, JG). Firstly, the titles and abstracts were analysed. Secondly, full-text articles were chosen for in-depth reading and analysed as per the inclusion and exclusion criteria for data extraction. All these above processes were done separately by the two reviewers, and then the level of agreement between the two reviewers, calculated by Cohen's kappa (*k*), was 0.94 for titles and abstracts and 0.96 for full texts. The differences among authors/reviewers were resolved by the third author (AMP) after discussion. For the clarification of doubts and missing data of the included studies, the respective authors were contacted by email if needed (none were contacted in our study).

### 2.5. Data Extraction

Two independent authors (SM, AML) extracted the following data independently and then correlated the data from the included studies. The data extracted was recorded under following headings: study identification number, authors, study design, follow-up, number of subjects, age, gender, method of education, oral radiology knowledge, radiographic interpretation skills, student's satisfaction, effect size, and author's remarks.

### 2.6. Assessments of the Risk of Bias and Quality

The level of evidence for every included study was assessed using the Joanna Briggs Institute (JBI) Levels of Evidence [15]. Risk of bias for the selected randomized controlled trials (RCTs) was executed by using the Cochrane Collaboration Tool [16] which including random sequence generation, allocation concealment, blinding of participants, incomplete outcome data, selective reporting, and other biases, while quality assessment of the same was done by the Agency for Healthcare Research and Quality (AHRQ) standard [17]. The quality assessment of nonrandomized studies was done using the MINORS checklist [18] with no restriction on the follow-up period being considered appropriate for the included studies, and the risk of bias was done using Risk of Bias in Nonrandomized Studies of Interventions (ROBIN-I) [19].

### 2.7. Statistical Analysis

Review Manager (RevMan) 5.3 was used for statistical analysis. The combined results were expressed as mean difference (MD) and standard deviation for the continuous data at 95% confidence intervals (CIs) and *P* < 0.05 was considered significant. Statistical heterogeneity was assessed by the *I*^2^ test at *α* = 0.10. For *I*^2^ > 50%, the random-effects model was applied. A funnel plot (plot of effect size versus standard error) was generated to examine possible publication bias.

## 3. Results

### 3.1. Literature Search

**Figure 1:**
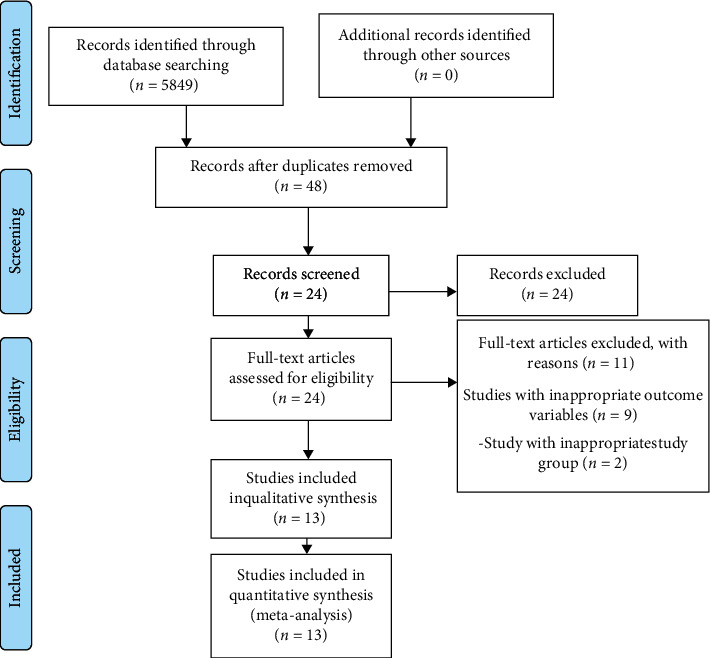
PRISMA flow diagram.

The PRISMA statement flowchart summarizing the selection process is shown in Figure 1. Among the 24 full-text articles, 13 were selected after prescreening, applying the eligibility criteria, and addressing the PICOS question. Eleven studies were excluded since 9 did not have appropriate outcome variables and 2 studies had an inappropriate study group; hence, only 13 studies were included in the qualitative and quantitative analysis.

### 3.2. Study Characteristics

There are 13 studies included in this review, the general characteristics of which are presented in Table 2. The studies included were conducted in different countries as follows: Australia [10], Brazil [20, 21], Greece [22], India [9, 12], North Carolina [23], South Korea [24], Sweden [25–27], and Toronto [8]. The study design of eight studies was randomized control trial [9, 10, 12, 23, 25–28] and the remaining five were nonrandomized studies [8, 20–22, 24].

All the participants included in the review were undergraduate dental students undertaking training in dental radiology [8–10, 12, 20–28]. A total of 835 participants were part of the studies' analysis, with 411 in the problem-based learning group and 424 in the traditional teaching group. All the participants already had the basic theoretical knowledge about the principles of radiographic interpretation and radiological features [8–10, 12, 20–28].

A significant methodological heterogeneity was observed with respect to the teaching methods described between the included studies. Hence, the teaching methods implemented by the included studies were categorized as follows: (1) problem-based learning methods—structured algorithm condition, smartphone-based training, case-based learning, syndicate learning, case-based blended learning, one-minute preceptor, small-group teaching, simulation teaching, virtual teaching, and schema-based teaching and (2) traditional teaching methods—the tutor-led method in which these radiographs were directly discussed by the tutor, basic science learning conditions, and traditional lectures. The problem-based and traditional teaching methods were used in the different studies that follow.

Soltanimehr et al. [25] conducted a study among the dental students to assess their theoretical knowledge and radiographic interpretation skills using a virtual teaching method which included the learning management system (LMS) that offers online and offline access of multimedia contents related to radiographic interpretations compared with traditional classroom lecture-based education over a period of 6 weeks. The virtual method was superior in improving the theoretical knowledge as compared to the traditional methods and had equal efficacy towards clinical reporting skills.

Ji et al. [24] conducted a study among third-year dental students to compare their level of satisfaction and overall self-awareness scores after the training program using smartphone-based training and the traditional lecture-based training in oral radiology. The smartphone-based training focused on provision of learning materials in advance via Google Classroom, schema assignments, group discussion activities, professor feedback, peer review, and tests (quizzes) for 1 week in groups of 10. Students in the control group who took part in traditional lectures participated in regular classes only. The authors concluded that even though the smartphone education with schema-based assignment was an attractive approach in oral radiology, the students showed lower levels of satisfaction with the same.

In a study by Busanello et al. [20], a digital learning object (DLO) method having two main sections comprising of radiopaque and radiolucent radiographic images of crown and root changes was evaluated. The images were also accompanied by hypertexts and clinical images for explanation as there was no presence of teachers. Sections with assignments and a quiz to empower students to practice and test their newly acquired knowledge were also added in DLO. The DLO was compared with conventional lectures to assess the radiographic diagnosing skills of undergraduate students. After 3 weeks with three 50 min classes per week, the study advocated that DLO was superior to the conventional teaching methods in improving the performance of the students.

Lohe and Singh [9] evaluated and compared effectiveness of syndicate learning with traditional learning for final BDS students in Oral Medicine and Radiology. In the syndicate learning method, students were given five radiographs having bony lesions for discussion, and they were free to use various resource materials like class notes, books, internet, etc., They had to complete the interpretation of the given radiographs by using the standard departmental reporting method in about 2 h during their clinical posting as well as present the report to the tutor who gave constructive feedback followed by active discussion. A tutor-led method in which the same five radiographs were directly discussed by the tutor was followed in the traditional learning method.

Naik and Umarani [12] assessed and compared the performances of the III BDS students in intraoral radiographic interpretation of periapical diseases using a structured checklist, namely, the objective structured radiographic interpretation (OSRI), after the one-minute preceptor and traditional training. The OMP group students were divided into small groups of six to seven students, and five different intraoral periapical radiographs of periapical diseases were discussed for a duration of 20 minutes. Then, the students interpreted the intraoral radiographs under the guidance of OMP principles for duration of one week. In the traditional training method, the students verbally interpreted the radiographs on a daily basis for a period of one week.

Baghdady et al. [8] assessed the effectiveness of nonanalytic strategies, i.e., making a diagnosis first and then identifying the radiographic features as compared to analytic strategies, i.e., identifying visual features first and then committing to a diagnosis using basic science instructions or a step-by-step algorithm among 2nd-year dental and dental hygiene students to test their diagnostic accuracy and memory retention skills immediately after learning and one week later. In the basic science learning condition, a causal explanation for the radiographic features along with its underlying pathophysiology were presented in its training material, whereas in the structured algorithm learning condition, the training included the same radiographic features but without the basic disease mechanism information.

Cruz et al. [21] assessed the immediate impact of distance learning using the Moodle platform by replacing it with traditional classroom learning for learning dental anatomy intraoral periapical radiographs. The traditional educational setting in a classroom was made by way of radiographic slabs, indicating anatomical structures, accompanied by a textbook describing the anatomical structure along with support from a teacher which was replaced by the digitalization of images and texts with a description of the anatomical structures related to imprints in images which were used to create the Moodle e-course.

A blended course which is a combination of face-to-face and online instruction was compared to conventional courses for its educational effectiveness on undergraduates in oral radiology by Kavadella et al. [22]. A blended course on differential diagnosis of mixed radiolucent-radiopaque bone lesions was developed, and its electronic version was uploaded to an e-learning educational platform. The students in the conventional group attended weekly lectures by the instructor, supported by PowerPoint presentations. Educational effectiveness of the course was determined by analysing the results of the knowledge-based questionnaires and the tests, which demonstrated that blended learning was effective as compared to conventional learning.

Sodestrom et al. [28] compared the influence of two learning conditions—a screen-based virtual reality radiology simulator and a conventional PowerPoint slide presentation—that teach radiographic interpretation to dental students working in small collaborative groups. The proficiency tests administered before and after training assessed interpretations of spatial relations in radiographs using parallax showing that the simulation-training group exhibited significant development as compared to the conventional group.

Vuchkova et al. [10] constructed a digital interactive learning tool consisting of PowerPoint presentation slides having text and labels denoting various anatomical features generally apparent in radiographs as an online resource to assist second-year dental students with their learning of radiographic anatomy and was compared to a conventional radiology textbook. All participants were then assessed on their understanding of radiographic anatomy. The results demonstrated improved quality of learning indicating strong preference for the digital interactive learning tool.

Nilsson et al. [26, 27]. conducted a study to assess the immediate as well as long-term effect of simulator-based training on the skill to interpret spatial information in radiographs as compared with conventional training. The simulator program was highly interactive having imaging and feedback qualities that cannot be performed in the real world. The participants in the experimental group trained individually using the simulator in two sessions of 45 minutes each. The control group used the ordinary educational material, consisting of cases with two or three intraoral radiographs accompanied by questions regarding projections and interpretation of spatial relations utilizing parallax with the tutor having a more active role in the control group.

The follow-up postintervention ranged from immediate assessment to about 8 months at maximum [ 8–10, 12, 20–28]. The drop out observed at follow-up ranged from 2.12% to 21% overall [ 8–10, 12, 20–28] (Table 2).

The outcome parameters assessed varying postinterventions across the studies. Except for two studies [22, 24], all the other eleven studies [8–10, 12, 20, 21, 23, 25–28] assessed radiographic interpretation skills by quantitative means using a radiographic interpretation test, proficiency score, and diagnostic accuracy scores; the knowledge score was assessed by four studies [20, 22, 24, 25]. Satisfaction scores were assessed by two studies [22, 24]. Overall, postintervention results favour improvements in the intervention groups in the assessed outcome parameters [8–10, 12, 20–28].

### 3.3. Assessments of the Level of Evidence, Risk of Bias and Quality

According to the JBI level of evidence, it appears that four studies [9, 23, 26, 27] were ranked at 1c, four studies [10, 12, 25, 28] as 1d, and the remaining five studies [8, 20–22, 24] as 2c (Table 3).

**Figure 2:**
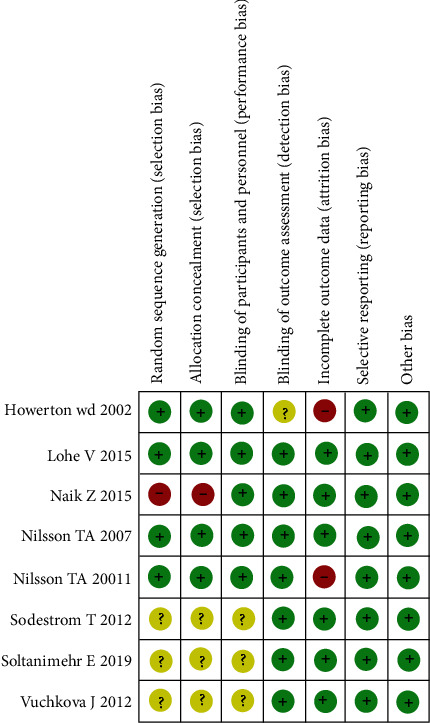
Risk of bias summary for the included studies.

**Figure 3:**
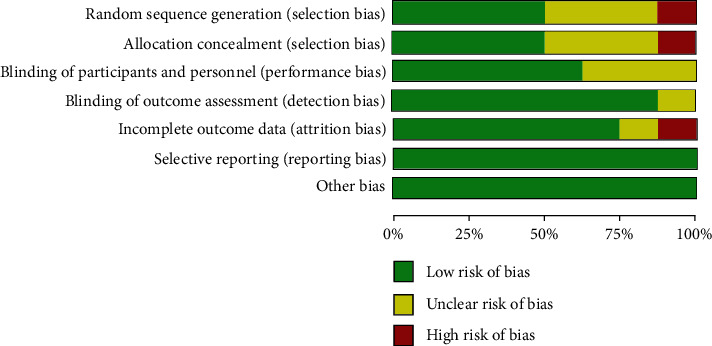
Risk of bias graph for all the included studies.

Quality assessment showed a huge variety across the included studies. Risk of bias of the eight randomized controlled trials (RCTs) was executed according to Cochrane Risk of Bias Tool [16] and quality assessment by the Agency for Healthcare Research and Quality (AHRQ) standard [17] (Table 4). Two studies [9, 27] showed a low potential risk of bias, four studies [10, 23, 25, 28] showed a moderate risk of bias, and two studies [12, 26] showed a high potential risk of bias (Figures 2 and 3). According to AHRQ, two studies [9, 27] showed good quality, one study [23] showed fair quality, and the remaining five [10, 12, 25, 26, 28] studies showed poor quality (Table 4).

The risk of bias of five nonrandomized studies was executed according to the ROBIN-I tool, where two studies [21, 24] showed low risk and the remaining three studies [8, 20, 22] showed moderate risk of bias (Table 5), whereas quality assessment was assessed using the methodological index for nonrandomized studies (MINORS) [18] for comparative studies wherein three studies [8, 20, 24] showed a score of 20 and the remaining two studies showed the score of 19 [22] and 22 [21] each. The detailed scores of the studies are presented in Table 6.

### 3.4. Synthesis of Results

**Figure 4:**
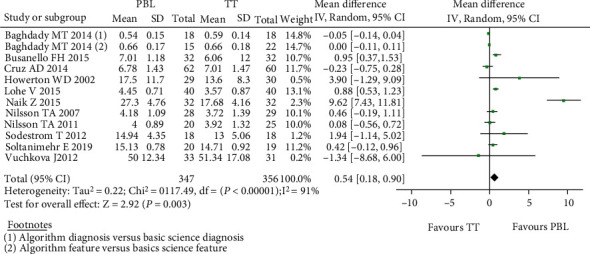
Forest plot for radiographic interpretation skills.

**Figure 5:**
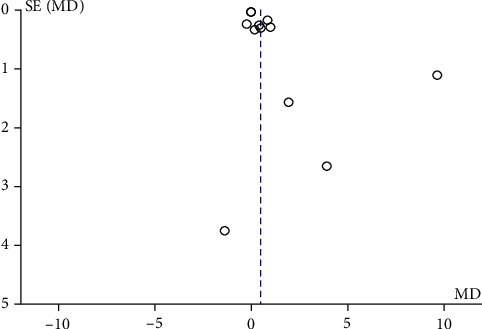
Funnel plot for publication bias for radiographic interpretation skills.

The studies that received any kind of PBL intervention vs. controls concerning the radiographic interpretation skills were analysed first. A study by Baghdady et al. [8] included 2 separate intervention and control groups which were analysed separately as two different studies. Therefore, on deducing the forest plot for twelve studies [8–10, 12, 20, 21, 23, 25–28], the mean difference in the proficiency score showed a positive difference of 0.54 (0.18, 0.90) with the random effect model based on the heterogeneity value of *I*^2^ which was statistically significant favouring the intervention group. Out of the twelve included studies in the forest plot for proficiency scores, nine studies [8, 9, 12, 20, 23, 25–28] showed positive mean difference values (Figure 4). A low risk for publication bias for this meta-analysis was indicated by the funnel plot (Figure 5).

**Figure 6:**
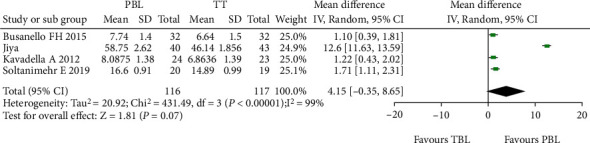
Forest plot of comparison knowledge scores.

**Figure 7:**
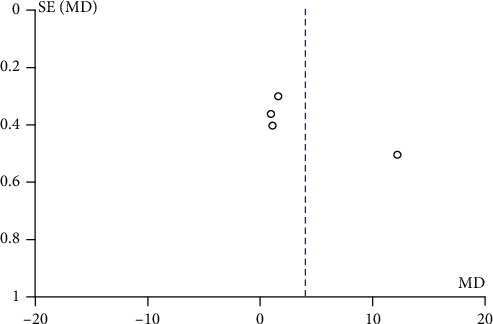
Funnel plot of publication bias for knowledge score.

The forest plot in Figure 6 demonstrates a significant difference in mean knowledge score between the PBL group and the TT group in all included studies assessed using the random effect model. The implant mean difference in the knowledge score for 116 and 117 students in the PBL and TT groups, respectively, in the included four studies [20, 22, 24, 25] was 4.15 with a minimum and maximum of -0.35 and 8.65, respectively. The funnel plot for this meta-analysis is displayed in Figure 7.

**Figure 8:**
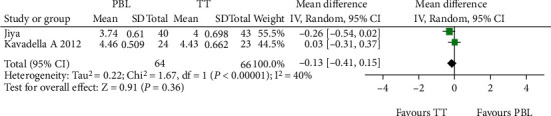
Forest plot of satisfaction level.

**Figure 9:**
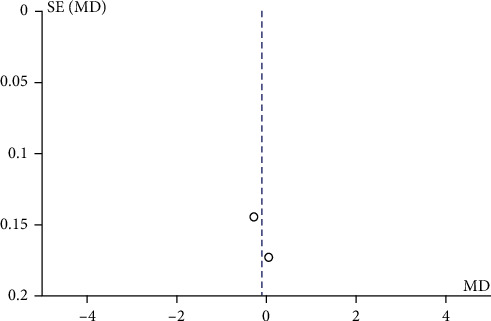
Funnel plot of publication bias for satisfaction level.

Figure 8 illustrates a forest plot showing a significant difference in satisfaction level favouring the TT group compared to the PBL group in the two included studies [22, 24] assessed using the random effect model. One study by Ji et al. [24] reported a negative change in the mean difference score of satisfaction level. Hence, the overall mean difference in the satisfaction level score showed a negative difference of -0.14 with a minimum and maximum of -0.36 and 0.08, respectively.  Figure 9 demonstrates a funnel plot indicating a low risk for publication bias for this meta-analysis.

## 4. Discussion

Through this systematic review and meta-analysis, the null hypothesis was rejected, thus indicating that there is a difference in PBL and traditional learning approach in improving acquisition of radiographic interpretation skills among dental students. Traditional lectures are still a common instructional method of delivering information verbally and are mainly a one-way method of communication that does not involve significant students' participation but relies upon passive learning. Lectures are useful in transmitting core knowledge and concepts especially to a large audience, but due to their nontransactional nature, they do not assess learning, offer varied perspectives, differentiate instruction, or allow students to self-direct [29]. But, the evolving methods of student learning necessitate the evolution of teaching methods [30]. The problem-based learning approach is an active learning method that fosters a variety of skills like teamwork, information finding, discussions, explanation of new information, and decision making among the students [29].

Dental radiology clinical practice mainly consists of radiograph acquisition and image interpretation centred on real clinical cases [24]. Varying approaches adopting the PBL objectives have been implemented in teaching oral radiology and are compared to the passive, teacher-centred traditional teaching approaches.

This systematic review and meta-analysis revealed the different kinds of problem-based learning approaches, which were mainly short-term interventions [8–10, 12, 20–25, 27, 28], except for one study [26] which was found to extend for 8 months. This review selected interventions targeted towards teaching oral radiology wherein all the students already had the basic theoretical knowledge about the principles of radiographic interpretation and radiological features and differential diagnosis [8–10, 12, 20–28]. To run any program of robust nature, funding plays an important role. Of the 13 studies included in the review, only four studies had international funding and collaboration [25–28], while the remaining nine studies were self-funded [25–28]. Ethical approval of a research project also helps to increase the legitimacy of research findings as well as plays a vital role in decision making based on the research results. Ten included studies in this review have mentioned about taking institutional ethical approval before the start of their study [8–10, 12, 20, 23, 25–28].

In the present systematic review, the educational effectiveness of the included studies [8–10, 12, 20–28] was assessed based on (i) the students' radiographic interpretation skills through mean proficiency and diagnostic accuracy scores, (ii) the students' performance through the knowledge tests, and

(iii) the level of satisfaction through the PBL methods; except in the study conducted by Ji et al. [24], only surveys on self-awareness of competency were conducted, and the students' achievement in terms of their true competency were not evaluated. Validity refers to how accurately a method measures what it is intended to measure. To obtain useful results, the methods and instruments used to collect data must be valid which ensures that the discussion of the data and the conclusions drawn are also valid. Twelve out of thirteen studies have tested the validity of the instrument used for assessing the outcomes [ 8–10, 12, 20–23, 25–28]. Without a doubt, all the included studies [8–10, 12, 20–28] have successfully accomplished their study objectives.

For the radiographic interpretation skills, twelve studies [8–10, 12, 20, 21, 23, 25–28] were included in the meta-analysis showing a significant difference favouring experimental groups. The introduction of new problem-based learning methods such as structured algorithm [8], syndicate learning [9], OMP [12], web-based learning [21], simulation [26–28], and virtual-based learning [10, 20, 23, 25] effectively facilitated student's exploration and self-study along with improved critical thinking as compared to traditional didactic. Also, the absence or passive role of tutors in the groups enabled students to take more responsibility for their own learning which was translated into improved radiographic interpretation skills.

For the knowledge score outcomes, four studies [20, 22, 24, 25] were included in the meta-analysis showing a significant difference favouring experimental groups. This improvement might be because of implementation of active learning methods that organized and systematized knowledge over memorization-based education, thus highlighting the importance of nonlinear education; encouraging students to favour self-directed, engaging learning; and confirming their clinical reasoning and problem-solving through schema-based assignments [24], blended learning [22], digital object learning [20], and virtual learning [25]. Also, a higher level of satisfaction was observed among the learners with improved learning because of easy and greater access to educational content via a virtual learning environment.

For the satisfaction level outcomes, two studies [22, 24] were included in the quantitative synthesis showing a significant difference favouring the traditional teaching group, which was controversial to other studies which were not included in this meta-analysis due to inappropriate outcome measures. The study conducted by Ji et al. [24] reported less satisfaction with respect to their interest in participation in schema-based learning as compared to those who received traditional training; this was because the students were unaccustomed to smartphone-based training, and the lack of immediate feedback by the professor made them lose interest in schema-based learning. In the study conducted by Kavadella et al. [22], it was reported that the students felt that blended learning was more demanding and was much more work than conventional training.

This systematic review included both randomized [9, 10, 12, 23, 25–28] and nonrandomized controlled trials showing heterogeneity in the included studies. Only four studies [9, 21, 24, 27] showed low risk of bias, whereas per AHRQ for randomized controlled trial, only two studies [9, 27] out of eight showed good quality, while five studies [10, 12, 25, 26, 28] were of poor quality due to inadequate random sequence generation, allocation concealment, and blinding of participants and personnel. In all the RCTs included [9, 10, 12, 25–28], except for one by Howerton et al. [23], the outcome assessment was done using objective assessment tools where the investigator had a passive role; hence, blinding of outcome assessment showed low risk of bias. However, in majority of the studies included, the sample size was not estimated leading to small sample sizes and decreased power of study. Also, a loss to follow-up of more than 5% was observed in four studies [8, 23, 24, 26] with 21% being the maximum. In publication bias, all of the funnel plots presented represent smaller studies with no beneficial effects. The methodological quality of majority of the studies is low except for few studies reporting radiographic interpretation skills which indicate absence of bias with replicable methodology (Figures 5, 7, and 9).

The present review has some limitations. It was not possible to fully avoid the clinical heterogeneity among the included studies. The sample size of the studies was small, thus lacking statistical power. Furthermore, the studies were conducted while the students were unused to the new method, which exerted a significant influence on their satisfaction levels. Also, there is a need for more trained staff for the tutoring process and learning the new methods of PBL. Students must relearn how to learn and teachers must relearn how to teach [4, 24]. Individual teaching method-wise analysis was not taken into consideration in the analysis. The subgroup and sensitivity analyses could have been performed to rule out the potential reasons for heterogeneity; however, this was not possible as there were small number of studies included under similar outcomes. There is no long-term evidence on the effectiveness of these interventions in oral radiology in improving radiographic interpretation skills, knowledge scores, and satisfaction levels among dental students. This may be due to the variability of PBL methods deployed in the individual studies.

In order to understand whether or not PBL methods are superior to traditional teaching methods in oral radiology among dental students, well-designed long-term randomized controlled trials are needed. This study was planned with a systematic review and a meta-analysis as these are both highly effective at analysing studies conducted on similar topics [31, 32]. Systematic reviews are efficient in quality evaluation of studies, while a meta-analysis is an objective method to carry out statistical analysis of various studies depending on their quality.

## 5. Conclusion

In conclusion, there was a difference in the evidence gathered showing that there is a difference between PBL and TL approaches and indicating that problem-based learning methods were effective in improving radiographic interpretation skills and knowledge scores over a short period. PBL was not effective for improving the satisfaction level among the students where the findings were conflicting. However, PBL fostered activation of prior learning, high motivation to learn, and the development of self-directed learning skills among the dental students.

## Figures and Tables

**Table 1:** The search strategy and PICOS tool.

*Search strategy*	
Focused question	Is there a difference in the effectiveness of problem-based learning (PBL) versus traditional teaching (TT) methods in improving acquisition of radiographic interpretation skills among dental students?
*Search strategy*	
Population	(Dental students [MeSH] OR dental undergraduate students [text word] OR undergraduate students [text word] OR dentistry students [text word] OR post graduate students [text word] OR students [text word] OR bachelor of dental surgery [text word])
Intervention	(Problem-based learning [MeSH] OR syndicate learning [text word] OR blended learning [text word] OR schema-based learning [text word] OR smartphone use [text word] OR experiential learning [text word] OR active learning [text word] OR problem based curricula [text word] OR one minute preceptor [text word] OR simulation-based learning [text word] OR conventional training [text word])
Comparisons	(Lecture [MeSH] OR instructional learning [text word] OR instructional method [text word] OR traditional clinical training [text word] OR traditional didactic method [text word])
Outcomes	(X-ray image [text word] OR dental X-ray [text word] OR X-ray diagnosis [text word] OR oral radiography [text word] OR dental radiography [text word] OR radiographic image interpretation [text word] OR interpretation skills [text word] OR diagnostic accuracy [text word] OR dental X-ray diagnostic accuracy [text word] OR dentomaxillofacial radiology [text word] OR radiographic image interpretation [text word])
Study design	(Clinical trials [MeSH] OR randomized controlled studies [text word] OR randomized control trials [MeSH] OR randomized control clinical trial MeSH OR non-randomized control trials [text word] OR quasi experimental studies [text word] OR before and after study design [text word] OR cohort studies [text word] OR in vivo study [text word])
Search combination	#1 AND #2 AND #3 AND #4
*Database search*	
Language	No restriction (articles in English language or other language where English translation is possible.)
Electronic databases	PubMed/MEDLINE, Cochrane Central Register of Controlled Trials, Web of Science
Journals	Dentomaxillofacial Radiology, European Journal of Dental Education, Journal of Contemporary Medical Education, BMC Medical Education, Journal of Dental Education
Period of publication	1-1-2000 to 30-06-2020

**Table 2:** Characteristics of the included studies.

Sr. no.	Study Id	Place of study	Study setting	Study design	Sample size	Total sample at follow − up = *N* (drop out %)	Population	Method of OHE for intervention group	Method of DHE for control group	Reinforcement period	Follow-up period	Method of outcome assessment	Diagnostic accuracy/radiographic interpretation skills/proficiency score	Knowledge scores	Recall test	Satisfaction with the training	Authors' conclusions
1.	Baghdady MT et al., 2014	Toronto	University	Nonrandomized trial	Test group: 40 (22 and 18) Control group: 40 (22 and 18)	Test group: 33 (15 and 18) 17.5% Control group: 40 (22 and 18) 0% Total loss to follow-up-8.75%	Second-year students at the University of Toronto and second-year dental hygiene students from a community college dental hygiene program	Structured algorithm condition: 1. Algorithm diagnosis 1st 2. Algorithm feature 1st	Basic science condition: 1. Basic science diagnosis 1st 2. Basic science features 1st	Once at baseline	1 week	1. Diagnostic test (mean/SD) 2. Cued recall test (mean/SD)	Baseline: *Test group* 1. Algorithm diagnosis 1st—0.69 (0.19) 2. Algorithm feature 1st—0.60 (0.15) *Control group* 1. Basic science diagnosis 1st—0.69 (0.18) 2. Basic science features 1st—0.61 (0.16) 1 week: *Test group* 1. Algorithm diagnosis 1st—0.66 (0.17) 2. Algorithm feature 1st—0.54 (0.15) *Control group* 1. Basic science diagnosis 1st—0.66 (0.18) 2. Basic science features 1st—0.59 (0.14)		Baseline: *Test group* 1. Algorithm diagnosis 1st—0.69 (0.19) 2. Algorithm feature 1st—0.60 (0.15) *Control group* 1. Basic science diagnosis 1st—0.69 (0.18) 2. Basic science features 1st—0.61 (0.16) 1 week: *Test group* 1. Algorithm diagnosis 1st—0.72 (0.21) 2. Algorithm feature 1st—0.77 (0.08) *Control group* 1. Basic science diagnosis 1st—0.74 (0.08) 2. Basic science features 1st—0.72 (0.09)		Students who learned the basic science mechanisms underpinning a disease might be more likely to make a diagnosis that made sense and not rely solely on counting the number of identifiable features on the image. This instructional methodology is in line with a nonanalytical reasoning strategy, in which the student would make a holistic diagnosis based on the totality of the identified features. Thus, left to their own devices, students who learn through basic science instruction should be more likely to use a nonanalytic reasoning diagnostic strategy. Participants in the diagnosis-first condition (nonanalytic reasoning) had higher diagnostic accuracy than those in the features-first condition (analytic reasoning), regardless of their learning condition.
2.	Busanello et al., 2015	Brazil	Dental Radiology Discipline of the Dentistry School	Nonrandomized trial	Test group—32 Control group—32	No loss to follow-up Total loss to follow-up 0%	Students enrolled in the dental radiology discipline of the dentistry school	Digital learning object (DLO) without the presence of a teacher	Conventional expository classes conducted by a teacher	Three 50 min classes were held per week, for 3 weeks	3 weeks	Knowledge scores (written scores) Diagnosis accuracy (practical scores only)	Posttest: Test group—7.01 (1.18) Control group—6.06 (1.20)	Posttest: Test group—7.74 (1.40) Control group—6.64 (1.50)			The results obtained in this study suggest that students who used the DLO performed better than those who used conventional methods. This suggests that the DLO may be a useful teaching tool for dentistry undergraduates, on distance learning courses and as a complementary tool in face-to-face teaching.
3.	Cruz AD et al., 2014	Brazil	Department of oral diagnosis	Nonrandom trial	AC—60 BC—62	No loss to loss-up Total loss to follow-up 0%	First and second semesters of 2011	“B class” (BC) —distance learning using the Moodle platform	“A class” (AC)—traditional method		Immediately after course completion	Radiographic interpretation scores	Posttest: BC—6.78 (1.43) AC—7.01 (1.47)				The method of distance learning of this subject using the Moodle platform can be utilized with the same educational results as those obtained from a traditional educational setting.
4.	Howerton WB et al., 2002	North Carolina	University of North Carolina School of Dentistry	Intervention study with posttest with controls	Group 1—34 Group 2—34	Group 1—30 (11.7) Group 2—29 (14.7) Total loss to follow-up 13.2%	First-year dental students, graduating class 2004, enrolled in “Fundamentals of Dental Radiology”	Group 2—students exposed to computer-assisted instruction before exposing the initial full mouth series. An interactive computer-assisted instructional module on CD.	Group 1—students not exposed to computer-assisted instruction before exposing the initial full mouth series	Group 2—no restrictions were placed on the number of times the CD could be viewed, and students were reminded several times by email to view the CD	One week	Total error points	Postintervention: Group 2: 17.5 (11.7) Group 1: 13.6 (8.3)				Students who received an interactive CAI CD before exposing their initial full series of radiographs made more errors than those students who did not receive the CAI CD. However, those students who received the CAI CD preferred reviewing the CD and recommended the CAI CD to others.
5.	Ji et al., 2018	South Korea	Dental school	Nonrandomized trial, posttest only with controls	Test group: 40 Control group: 43	Test group: 35 (12.5%) Control group: 42 (2.3%) Total loss to follow-up 7%	Third-year students in Wonkwang Dental College	Smartphone-based training—comprised of the provision of learning materials in advance, schema assignments, group discussion activities, professor feedback, peer review, and tests (quizzes)	Traditional lecture-based training	Test group—received focused lectures for 1 week (5 days) in groups of 10 (only 1 turn in 4 weeks) Control group—received lectures for a time per 1 week (4 lectures in 4 weeks)	4 weeks	1. Satisfaction with the training 2. Quiz results		4 weeks Test group: 58.75 (2.62) Control group: 46.14 (1.856)		4 weeks Test group: 3.74 (0.610) Control group: 4.00 (0.698)	The dental radiology schema education using smartphones suggested in the present study is not a method often used in dental education, and its effects have not been verified. Nevertheless, the training requires the interest of dental educators of the current generation as a new teaching method that could be introduced in preparation for the fourth industrial revolution for dentomaxillofacial radiology practice.
6.	Kavadella A et al., 2012	Athens, Greece	School of Dentistry of the University of Athens	Nonrandomized trial—pre-post-test with controls	Test group—24 Control group—23	Test group—24 (0%) Control group—22 (4.3%) Total loss to follow-up—2.12%	10th semester (final year) in the School of Dentistry of the University of Athens	Blended group—combined face-to-face and online instruction	Conventional group	Weekly till end of course	Not mentioned	1. Students' attitudes postcourse 2. Knowledge score pre- and postcourse		*Pretest*: Blended group—6.3583 (1.21) Conventional group—5.8304 (1.58) *Posttest*: Blended group—8.0875 (1.38) Conventional group—6.8636 (1.39)		*Posttest*: Blended group—4.46 (0.509) Conventional group—4.43 (0.662)	Concerning student performance, students in the blended group performed significantly better in the knowledge posttest than their colleagues in the conventional group. Students also evaluated the course components in a positive way: the content, organization, educational material, and design were highly appreciated by students in both groups. Students' attitudes towards blended courses were positive: they think that blended learning is effective and motivating; it promotes active engagement and enhances self-study and self-assessment. Particularly, students in the blended group liked the combination of electronic and face-to-face teaching, the independent studying, and the availability of the online material at any time.
7.	Lohe V et al., 2015	Wardha, India	The Department of Oral Medicine and Radiology, Sharad Pawar Dental College	Randomized controlled trial	Syndicate group—40 Traditional group—40	No loss to follow-up Total loss to follow-up—0	Final BDS students	Group A—syndicate learning method by giving five radiographs having bony lesions for discussion. The students were free to use various resource materials like class notes, books, internet, etc. They had to complete the interpretation of the given radiographs by using the standard departmental reporting method in about 2 h during their clinical posting.	Group B—traditional learning method is a teacher-centred small group method wherein the students remain comparatively passive	Only once	Immediately after discussion	Interpretation skills score in pretest and posttest	*Pretest*: Syndicate group—2.40 (0.98) Traditional group—2.47 (0.96) *Posttest*: Syndicate group—4.45 (0.71) Traditional group—3.57 (0.87)				Syndicate groups create many opportunities for creative interchange of ideas and lively and meaningful participations. This approach would ensure that, in addition to gaining subject-specific knowledge, students are also able to apply the obtained knowledge to solve problems. The present study suggests that the syndicate group is better than the traditional method and can become an appropriate method as an adjunctive instruction tool.
8.	Naik Z et al., 2015	Karnataka, India	Department of Oral Medicine and Radiology	Randomized pre-post trial	Intervention group—32 Comparison group—32	No loss to follow-up Total loss to follow-up—0	Third-year BDS students	One-minute preceptor group—students were divided into small groups of six to seven students and five different intraoral periapical+M5 radiographs of periapical diseases were discussed for a duration of 20 minutes. Then the students interpreted the intraoral radiographs under the guidance of OMP principles	Traditional group—students verbally interpreted the radiographs on a daily basis	Daily	One week	Radiographic interpretation skills, pretest and posttest scores	*Pretest*: Intervention group—5.30 (2.24) Traditional group—5.32 (1.34) *Posttest*: Intervention group—27.30 (4.76) Traditional group—17.68 (4.16)				This study supports the critical role of the radiographic interpretation in enhancing diagnostic accuracy in oral radiology. It also supports the use of the OMP model for systematic radiographic examination as a possible explanation for significant improvement of radiographic interpretation skills in a stipulated time setting. Thus, by using the “one-minute preceptor” model, student's radiographic interpretation skills had progressed from unorganized and inconsistent to systematic and consistent with clinical diagnosis, thus achieving an important skill to be a competent general dental practitioner.
9.	Nilsson TA et al., 2011	Sweden	Oral and Maxillofacial Radiology Department at the University Clinic	Randomized experimental study	Experimental group—28 Control group—29	Experimental group—20 (19.6) Control group—25 (13.8) Total loss to follow-up 21%	The seventh and ninth semesters	Simulation-based training—the participants in the experimental group trained individually using the simulator. The leader introduced the exercises and thereafter only answered questions. During training, the students were free to choose among the exercises. Training was carried out in two sessions of 45 minutes each. The time interval between the two training sessions varied from 1 day to 2 weeks.	Conventional training—the training was completed in one 90-minute session	8 months	Immediately after training and 8 months	Students' skill in interpretation of spatial information in radiographs	*Pretest*: Experimental group—3.20 (1.40) Control group-3.40 (1.16) *Immediate*: Experimental group—4.15 (1.14) Control group—3.80 (1.35) *8 months*: Experimental group—4.00 (0.86) Control group—3.92 (1.32)				In conclusion, the skill of interpreting spatial relations after simulator-supported training was better eight months after training than before training. The conventional training showed a similar outcome pattern, but at a lower level. Simulator-supported training can therefore be a valuable adjunct to conventional educational methods.
10.	Nilsson TA et al., 2007	Sweden	Oral and Maxillofacial Radiology Department at the University Clinic	Randomized experimental study	Experimental group—28 Control group—29	No loss to follow-up Total loss to follow-up 0%	The seventh semester and ninth semester dental students	Simulation-based training—the participants in the experimental group trained individually using the simulator. The leader introduced the exercises and thereafter only answered questions. During training the students were free to choose among the exercises. Training was carried out in two sessions of 45 minutes each. The time interval between the two training sessions varied from 1 day to 2 weeks.	Conventional training—the training was completed in one 90-minute session.	No	Immediately after training	Proficiency test (radiography subtest)	*Pretest*: Experimental group—3.11 (1.42) Control group—3.24 (1.15) *Posttest*: Experimental group—4.18 (1.09) Control group—3.72 (1.39)				In conclusion, our study demonstrated that training in the radiology simulator improved skill at interpreting spatial information in radiographs utilizing parallax when evaluated immediately after training.
11.	Sodestrom T et al., 2012	Sweden	Umeå University	Randomized experimental study	Intervention group—18 Control group—18	No loss to follow-up Total loss to follow-up 0%	Fourth semester	Simulation-training group (SIM)	Conventional-training group (CON)	SIM group worked one hour with a 3D-radiology simulator to perform four structured exercises CON group studied for one hour using pairs of X-ray images shown in a PowerPoint presentation	Posttraining	Proficiency test	*Pretest*: SIM group—12.94 (4.29) CON group—13.11 (2.96) *Posttest*: SIM group—14.94 (4.35) CON group—13.00 (5.06)				The results showed that SIM groups exhibited significant development between pretest and posttest results, whereas the CON groups did not. The collaboration in the CON groups involved inclusive peer discussions, thorough interpretations of the images, and extensive use of subject-specific terminology. The SIM group discussions were much more fragmented and included more action proposals based on their actions with the simulator. The different learning conditions produced different results with respect to acquiring understanding of radiographic principles.
12.	Soltanimehr et al., 2019	Iran	Shiraz University, School of Dentistry	Experimental study	Intervention group—20 Control group—19	No loss to follow Total loss to follow-up 0%	Fourth-year dental students	Virtual group—learning management system (LMS), which included a combination of facilities such as a learning path, quizzes, weekly homework, useful links, related articles, and active interactions of students and mentors.	Traditional group—lecture-based education in a classroom setting in the presence of a mentor	Virtual group—group of students were allowed to use the LMS repeatedly during 6 weeks Traditional group—6 sessions of traditional classroom instruction, 1 h each	Immediately and 2-month follow-up	Theoretical knowledge scores clinical exams scores	*Immediate clinical exam*: Virtual learning: 15.13 (0.78) Traditional learning: 14.71 (0.92) *Clinical exam at 2 months*: Virtual learning: 14.75 (0.87) Traditional learning: 14.18 (0.95)	*Immediate theoretical tests*: Virtual learning: 16.60 (0.91) traditional learning: 14.89 (0.99) *Theoretical tests at 2 months*: Virtual learning: 15.88 (0.78) Traditional learning: 14.45 (0.83)			The virtual method was more effective than the traditional method for instruction of radiographic interpretation of bony lesions of the jaw. However, this superiority was greater for the theoretical aspect of the topic. Considering the superiority of the virtual method for teaching of theoretical topics and its equal efficacy with the traditional method for instruction of clinical reporting skills, virtual education can serve as an effective alternative to traditional classroom teaching for teaching of radiographic interpretation of bony lesions of the jaw to dental students.
13.	Vuchkova J et al., 2012	Australia	School of Dentistry at the University of Queensland	Experimental study	Group B—33 Group A—31	No loss to follow-up Total loss to follow-up 0%	Second-year undergraduate dental students	Digital tool	Conventional oral radiology textbook	Each group underwent a 1 h intervention phase involving the learning of radiographic anatomy	Immediately postintervention	Radiographic interpretation scores	*Postintervention*: Group B: 50.00 (12.34) Group A: 51.34 (17.08)				Although the newly constructed digital tool was not quantitatively superior to the conventional textbook in assisting dental students with their learning of radiographic interpretation, qualitative measures indicated a strong preference for the digital tool as a learning and teaching resource in radiographic interpretation.

**Table 3:** Level of evidence according to JBI levels of evidence.

Sr. no.	Study ID	Level of evidence
1.	Baghdady MT et al., 2014	2c
2.	Busanello FH et al., 2015	2c
3.	Cruz AD et al., 2014	2c
4.	Howerton WB et al., 2002	1c
5.	Ji YA et al., 2018	2c
6.	Kavadella A et al., 2012	2c
7.	Lohe V et al., 2015	1c
8.	Naik Z et al., 2015	1d
9.	Nilsson TA et al., 2011	1c
10.	Nilsson TA et al., 2007	1c
11.	Sodestrom T et al., 2012	1d
12.	Soltanimehr E et al., 2019	1d
13.	Vuchkova J et al., 2012	1d

**Table 4:** Risk of bias and quality assessment for randomized controlled trials.

Sr. no.	Study ID	Random sequence generation	Allocation concealment	Blinding of participants and personnel	Blinding of outcome assessment	Incomplete outcome data	Selective reporting	Others	Overall risk of bias	Agency for Healthcare Research and Quality (AHRQ) standard
1.	Howerton WB et al., 2002	Low	Low	Low	Unclear	Unclear	Low	Low	Unclear risk	Fair quality
2.	Lohe V et al., 2015	Low	Low	Low	Low	Low	Low	Low	Low risk	Good quality
3.	Naik Z et al., 2015	High	High	Low	Low	Low	Low	Low	High risk	Poor quality
4.	Nilsson TA et al., 2011	Low	Low	Low	Low	High	Low	Low	High risk	Poor quality
5.	Nilsson TA et al., 2007	Low	Low	Low	Low	Low	Low	Low	Low risk	Good quality
6.	Sodestrom T et al., 2012	Unclear	Unclear	Unclear	Low	Low	Low	Low	Unclear risk	Poor quality
7.	Soltanimehr E et al., 2019	Unclear	Unclear	Unclear	Low	Low	Low	Low	Unclear risk	Poor quality
8.	Vuchkova J et al., 2012	Unclear	Unclear	Unclear	Low	Low	Low	Low	Unclear risk	Poor quality

**Table 5:** Risk of bias judgement for nonrandomized trials (ROBIN-I tool).

Bias domain	Baghdady MT et al., 2014	Busanello FH et al., 2015	Cruz AD et al., 2014	Ji YA et al., 2018	Kavadella A et al., 2012
Bias due to confounding	N	N	N	N	N
Bias in selection of participants into the study	N	PN	N	N	PN
Bias in classification of interventions	N	N	N	N	N
Bias due to deviations from intended interventions	N	N	N	N	N
Bias due to missing data	PN	N	N	N	N
Bias in measurement of outcomes	N	N	N	N	N
Bias in selection of the reported result	N	N	N	N	N
Overall bias	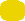	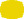	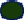	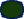	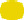

Green circle=low risk; yellow circle=moderate risk; red circle=high risk; N=number; PN=partial number.

**Table 6:** Methodological index for nonrandomized studies (MINORS).

	A clearly stated aim	Inclusion of consecutive patients	Prospective collection of data	Endpoints appropriate to the aim of the study	Unbiased assessment of the study endpoint	Follow-up period appropriate to the aim of the study	Loss to follow-up less than 5%	Prospective calculation of the study size	^∗^An adequate control group	^∗^Contemporary groups	^∗^Baseline equivalence of groups	^∗^Adequate statistical analyses	Total
Baghdady MT et al. (2014) [8]	2	2	2	2	2	2	0	0	2	2	2	2	20
Busanello FH et al. (2015) [20]	2	1	2	2	2	2	2	0	2	2	1	2	20
Cruz AD et al. (2014) [21]	2	2	2	2	2	2	2	0	2	2	2	2	22
Ji YA et al. (2018) [24]	2	2	2	2	2	2	0	0	2	2	2	2	20
Kavadella A et al. (2012) [22]	2	0	2	2	1	2	2	0	2	2	2	2	19

^†^The items are scored 0 (not reported), 1 (reported but inadequate), or 2 (reported and adequate). The global ideal score is 16 for noncomparative studies and 24 for comparative studies. ^∗^For study with control group.

## Data Availability

All data used to support the findings of this study are included within the article.
